# Global climate changes drive ecological specialization of mammal faunas: trends in rodent assemblages from the Iberian Plio-Pleistocene

**DOI:** 10.1186/1471-2148-13-94

**Published:** 2013-04-30

**Authors:** Ana R Gómez Cano, Juan L Cantalapiedra, Aurora Mesa, Ana Moreno Bofarull, Manuel Hernández Fernández

**Affiliations:** 1 Departamento de Paleontología, Facultad de Ciencias Geológicas, Universidad Complutense de Madrid, José Antonio Novais 2, Madrid 28040, Spain; 2 Departamento de Paleobiología, Museo Nacional de Ciencias Naturales, Consejo Superior de Investigaciones Científicas, Pinar 25, Madrid 28006, Spain; 3 Departamento de Cambio Medioambiental, Instituto de Geociencias (UCM, CSIC), José Antonio Novais 2, Madrid 28040, Spain

**Keywords:** Biome, Community, Evolutionary ecology, Generalist, Glaciations, Habitat theory, Macroevolution, Specialist

## Abstract

**Background:**

Several macroevolutionary hypotheses propose a synchrony between climatic changes and variations in the structure of faunal communities. Some of them focus on the importance of the species ecological specialization because of its effects on evolutionary processes and the resultant patterns. Particularly, Vrba’s turnover pulse hypothesis and resource-use hypothesis revolve around the importance of biome inhabitation. In order to test these hypotheses, we used the Biomic Specialization Index, which is based on the number of biomes occupied by each species, and evaluated the changes in the relative importance of generalist and specialist rodents in more than forty fossil sites from the Iberian Plio-Pleistocene.

**Results:**

Our results indicate that there was a decrease in the specialization degree of rodent faunas during the Pliocene due to the global cooling that triggered the onset of the glacial events of the Cenozoic (around 2.75 Ma). The subsequent faunal transition after this critical paleoenvironmental event was characterized by an increase of specialization related to the adaptation to the new environmental conditions, which was mainly associated with the Pleistocene radiation of Arvicolinae (voles).

**Conclusions:**

The pattern of faunal turnover is correlated with the development of the modern glaciations in the Northern Hemisphere around 2.75 Ma, and represents a reorganization of the rodent communities, as suggested by the turnover pulse hypothesis. Our data also support the resource-use hypothesis, which presumes the role of the degree of specialization in resources specifically related to particular biomes as a driver of differential speciation and extinction rates. These results stress the intimate connection between ecological and evolutionary changes.

## Background

Almost since the first publication of the seminal contribution about the tendency of species to form varieties [1], there has been a debate between models that consider the competition between species as the key for evolutionary changes [2,3] and the ones that regard factors external to the species, usually biogeography or climatic changes, as the main drivers responsible of biotic evolution [4-7]. Nevertheless, an increasing consensus has appeared during the last decades on the critical importance of changes in the physical environment, rather than biotic interactions themselves, for the evolution of organisms and ecosystems at large spatial and time scales [8-11].

The habitat theory proposed by Elisabeth S. Vrba [12,13] is one of the best known of such evolutionary models. This theory comprises a set of hypotheses showing the influence of global climatic shifts and the subsequent environmental changes on the turnover of species assemblages as a result of the drifting of geographic distributions, lineage originations and extinctions.

As a core part of the habitat theory, Vrba [7,14] developed the resource-use hypothesis, which stresses the relationship between the ecological specialization of species and the processes that regulate their evolution. The different conditions imposed by the ecological characteristics of species, and especially the biomes that they inhabit, have an effect on the macroevolutionary patterns that are observed through time and space [7,14-17]. In this way, the resource-use hypothesis gives a great value to the differences between the species that inhabit one biome exclusively (biome specialists or stenobiomic species) and others that obtain their resources from more than one biome (biome generalists or eurybiomic species). In order to clarify the concepts used for this work, it is important to indicate that biome specialization is not necessarily related to specialization in other ecological traits of the species. For example, *Rhynchomys soricoides* (Murinae, Rodentia), which has been described as vermivore [18], exhibits a high grade of specialization in its dietary habits but, since it inhabits three different biomes (equatorial rainforest, tropical deciduous woodland and temperate evergreen forest) [19-21], is a biome generalist. On the other hand, *Stochomys longicaudatus* (Murinae, Rodentia) has an omnivore diet [22], but it is restricted to the equatorial rainforest of Central Africa [23].

According to the resource-use hypothesis, during episodes of climatic triggering of habitat change, specialist species are more prone to suffer limitation of their resources and, consequently, they are more susceptible to habitat fragmentation, vicariance and directional selection. Since environmental changes have stronger effects on biome specialists than on generalist species, which can find their resources in different biomes, the former are predicted to have higher speciation and extinction rates than the latter [14,17]. Such phenomenon results in an increase of the specialist species versus generalists in the global fauna, which has been observed in mammalian assemblages from both Africa [14,15] and South America [16] as well as in the ruminants at the global scale [17].

Importantly, because of the constraints on the evolutionary history of species imposed by changes in the physical environment, most of the evolutionary changes in biotic lineages (including speciation, extinction or dispersion) should occur synchronically with global climatic changes. Such an evolutionary scenario is developed in the turnover-pulse hypothesis [12,24,25]. According to this hypothesis, most speciations and extinctions across diverse groups of organisms are not randomly distributed in time, but show statistically significant concentrations near times of major physical change. While most of these turnover-pulses affect few lineages and/or restricted geographic areas, some of them are massive and of global extent [25].

**Figure 1:**
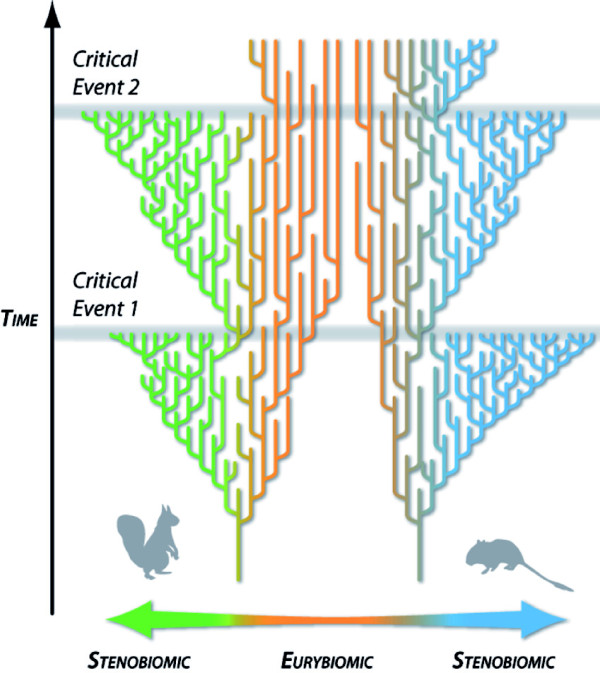
**Schematic representation of the turnover-pulse and the resource-use hypotheses.** Iterative and differential evolution of clades related to variations in biomic specialization as predicted by the turnover-pulse and the resource-use hypotheses (modified from Vrba [25,26]). During severe environmental changes, extinction rates of stenobiomic lineages reach their apex, giving rise to critical biotic events in which generalist species constitute most of the survivors. After each critical event the development of new specialist lineages with faster speciation rates generates specialists-dominated faunas as the environmental conditions are stabilized.

The combination of all these issues, along with the hierarchical organization of ecological and evolutionary processes [26], raises three corollary predictions (Figure 1): (1) due to their large ecological preferences, biome generalists might constitute a predominant part of the set of species that survive during the moments of significant global change; (2) nevertheless, once the critical periods of global change have finished, the preponderance of generalist species will decrease and a set of new specialist species will develop through speciation of the surviving lineages as the environmental conditions are stabilized; (3) this would entail a progressive specialization of generalist clades through niche filling within the newly generated environments. On the other hand, due to ecological constraints, clades dominated by specialist species before the crisis are expected to severely decrease in importance, although their specialization degree may not change.

To test these predictions, here we focus on the rodent faunas from the Iberian Plio-Pleistocene. During this period, successive cooling pulses culminated with the establishment of continental northern-hemisphere glaciations and the modern ice age climate around 2.75 Ma [27-29]. This severe climatic event at the global scale is an ideal scenario for testing Vrba’s hypotheses. We used rodents as our study group because they are widespread, highly diverse and habitat-sensitive, which makes them one of the most environmental and climatically informative groups of mammals [30-34]. Besides, their Iberian fossil record is vast and extensively documented [35]. Additionally, this group has been used to characterize intervals of great faunal change throughout the Cenozoic, usually associated with global climate fluctuations (e.g. [36,37]). For these reasons they provide a suitable faunal set to test the predictions exposed above.

## Methods

### Data

In this work we used the faunal lists of rodent communities from 44 fossil sites from the Iberian Plio-Pleistocene (Table 1) dated between 5.25 and 0.01 Ma [38]. These fossil sites have been subject to intensive sampling during the last fifty years (see references in [38]). The minimum sample required to include a fossil site in our study was 100 first and second upper and lower molars, which is considered the minimum number necessary to render a representative sample of the original paleocommunity [30,36,39].

**Table 1:** Fossil sites used in this work and values of average BSI

**Fossil sites**	Age (Ma) ^*^	**N **^**†**^	**Average BSI **^**§**^
Caldeirâo Eb	0.012	9	1.778
Caldeirâo Fa	0.012	7	1.429
Caldeirâo Fb	0.012	8	1.750
Cueva Millán 1a	0.038	7	2.800
Pinilla del Valle	0.191	14	2.231
Las Yedras	0.191	8	1.714
Cueva del Agua	0.266	8	2.286
Áridos 1	0.266	6	2.000
Galería III	0.340	11	2.100
Galería IIb	0.340	12	2.091
Galería IIa	0.340	12	2.182
Cueva de los Zarpazos 4	0.340	9	2.444
Trinchera Dolina 10	0.340	9	2.125
Cúllar Baza 1	0.430	5	2.400
Trinchera Penal Tubo 2	0.852	5	2.600
Trinchera Penal 8	0.852	7	2.000
Trinchera Penal 7	0.852	11	2.375
Trinchera Dolina 6	1.110	14	2.571
Trinchera Dolina 5	1.110	14	2.538
Trinchera Dolina 4	1.110	13	2.667
Trinchera Dolina 3	1.110	9	2.333
Huéscar 1	1.472	6	1.500
Sima del Elefante	1.472	11	2.375
Quibas	1.782	5	2.800
Bagur 2	1.782	8	2.143
Casablanca 1	2.040	8	2.429
Valdeganga III	2.144	6	2.500
Casablanca B	2.402	5	2.800
Huélago 5	2.557	4	2.333
Escorihuela A	2.971	9	3.000
Escorihuela	2.971	11	2.818
Sarrión	3.281	10	2.556
Moreda 1	3.281	18	2.625
Barranco de Quebradas 1	3.436	6	2.000
Layna	3.591	14	2.500
Orrios 1	3.746	8	1.857
Arquillo III	4.056	15	2.733
Aldehuela	4.263	9	1.857
Villalba Alta 1	4.263	15	2.333
Gorafe 1	4.521	11	1.625
Caravaca 1	4.728	10	1.333
Peralejos E	4.832	12	2.417
La Gloria 4	4.832	16	2.313
Purcal 4	5.245	11	2.000

^*^ Ages after [38].

^†^ N, number of species.

^§^ BSI, Biomic Specialization Index.

The specialization degree of each species was measured using the Biomic Specialization Index (BSI) developed by Hernández Fernández and Vrba [15]. This index indicates the number of biomes inhabited by the species, following the climatic classification of Walter [40], which recognizes ten biomes. Therefore, BSI equals 1 for most specialized species whereas generalist species could exhibit a BSI as high as 10. The data on the biome residence for all the rodent species were obtained from [34], who derived biome residences from identifying their living ecological analogues as estimated by ecomorphological studies of the dentition [41,42].

Finally, following Hernández Fernández and Vrba [43], we calculated the relative frequency of specialist and generalist species in each fossil site in terms of the average value of the BSI of the species found in the site. Taking into account that some taxa on our lists were not identified to species level, we decided to conduct the analysis by two different ways. First, for the undetermined taxa, the BSI value was calculated as the average of all the species that belong to their upper taxonomic level. Second, to avoid the potential noise in the data due to unidentified taxa, we only analysed the taxa that were determined at species level in each fossil site. Since both analyses yielded very similar results, here we only discuss those corresponding to the latter version of the analysis (all taxa at the species level).

### Analyses

Following our predictions derived from the interaction between the resource-use and the turnover-pulse hypotheses, the beginning of the modern glaciations in the Northern Hemisphere (around 2.75 Ma) should be coincident with the rising of mammal faunas dominated by generalists (high average BSI values). After this critical event we should find a progressive increase in the specialization degree of the rodent faunas from the Iberian Pleistocene (decrease of average BSI). In order to identify significant changes in the average BSI of fossil assemblages through time, we carried out Multivariate Adaptive Regression Splines (MARS; [44]) on the data set. MARS is a method that identifies hinge points in a time series that automatically minimize the residual sum of squares (RSS). In this way time shifts in BSI trends do not need to be fixed a priori. Progressively adding hinge points to the model increases the number of parameters. Since overly complex models may result in stochastic error (inflated variance), we need to find an equilibrium between fit and complexity. MARS does this automatically using the Akaike Information Criterion (AIC) scores of each model, which measures the goodness of the fit of a statistical model while penalizing the number of parameters (the complexity).

Additionally, we expected to find different evolutionary responses to this event in different rodent clades according to their degree of specialization. More generalist groups are predicted to survive and proliferate after the environmental crisis at 2.75 Ma, becoming progressively more specialized. Meanwhile clades dominated by specialist species before the crisis are expected to decrease in importance, although the specialization degree may not change. These predictions were tested by means of t-Student comparisons between species pools before and after the inflection point yielded by the MARS analysis within different taxonomic groups. Following Wilson & Reeder [45], we studied the specialization trend of Sciuridae, Gliridae, Castoridae, Arvicolinae, Cricetinae, Gebillinae, Murinae and Hystricidae separately. Subfamilies within Cricetidae (arvicolines and cricetines) and Muridae (gerbillines and murines) [19] were studied independently due to their importance in Iberian Plio-Pleistocene rodent faunas, and because they have been traditionally taken as independent families in paleontological studies [35] and their monophyly has been demonstrated by molecular studies [46,47].

## Results

**Figure 2:**
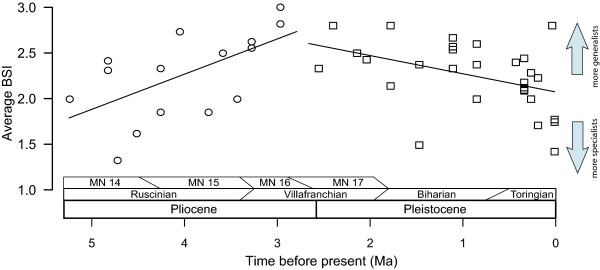
**Time series of average BSI for rodent faunal assemblages through the Iberian Plio-Pleistocene.** Average biomic specialization index (BSI) estimated for 44 fossil sites from the Iberian Plio-Pleistocene dated between 5.25 and 0.01 Ma. Solid and dashed lines are the results of the MARS analysis [44], which identified a hinge point (Table 2) in the trend that separates the assemblages before (circles) and after (squares) the onset of the modern glaciations in the Northern Hemisphere ~ 2.75 million years ago. The implied Mammal Neogene zones (MN, [48]), European land mammal ages (see [38]) and epochs are shown at the bottom.

Through the time span considered here, our results show the existence of two opposed patterns with an inflection point coincident with the onset of the Pleistocene glaciations. This involves substantial changes in the average BSI value of the Iberian rodent faunas (Figure 2), supporting the prediction of a change in the pattern of ecological specialization in concert with the cooling pulse of the Plio-Pleistocene around 2.75 Ma.

We observed a considerable increase of the average BSI throughout the Pliocene, reaching maximum values close to the end of the period. This is to say; during the Pliocene there was a transition from faunas with a higher prevalence of specialist species to faunas where biome generalists were predominant. The best-fit model identified by MARS analysis (Table 2) includes a single hinge point in the BSI time-series that divides the time series in two sub-sets differing in their trend. The first sub-set of data ranges from 5.3 Ma to 2.9 Ma, depicting an increase in BSI values (towards less specialists; see Figure 2). The second sub-set ranges from 2.6 Ma to the present and shows a decrease of the BSI values (towards more specialists; see Figure 2). Adding this hinge point to the model resulted in a significant improvement of the fitness over the null model where specialization (BSI) remains linear through time (*Δ*AIC = 13.28, see Table 2). Increasing the number of hinge points did not improve the fit of the models (Table 2).

**Table 2:** AIC scores

**Hinges**	**AIC**	***Δ*AIC**	**Time-shifts (Ma)**			
**0**	88.99	13.28	–			
**1**	75.71	0	2.97			
**2**	77.71	2	2.97	3.43		
**3**	79.71	4	2.40	2.97	3.43	
**4**	81.71	6	2.40	2.97	3.43	4.06

AIC scores of the models with different number of hinge points according to the MARS analysis [44]. The lowest AIC represents the best fit to the data. Age for each hinge is shown.

**Figure 3:**
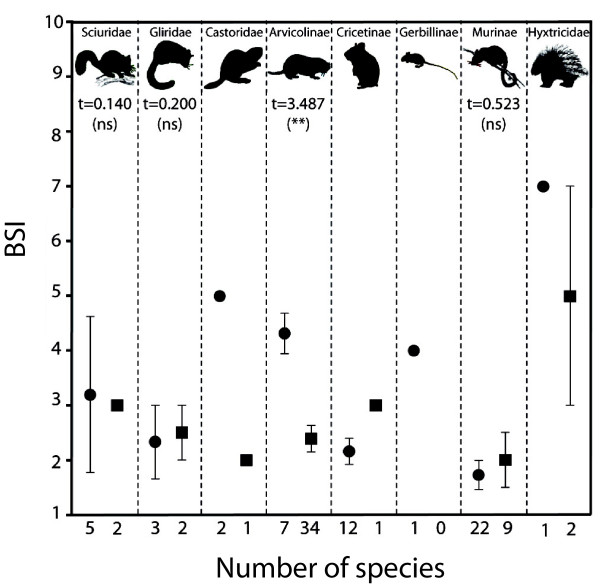
**Species BSI (average ± standard error) values for each rodent clade.** Species BSI (average ± standard error) values for each rodent clade in the Plio-Pleistocene record from the Iberian Peninsula. Values before (circles) and after (squares) the onset of the modern northern hemisphere glaciations, 2.75 million years ago, are shown. T-student tests for both assemblages in each clade are indicated when statistical comparisons were possible. **, p < 0.01; ns, non significant. Taxonomic groups are ordered according to Wilson and Reeder [45].

The t-student tests for different rodent groups indicated that the radiation of arvicolines (voles) is related to a significant change in biomic specialization, from few generalists to many specialist species, associated to the critical environmental change at 2.75 Ma (Figure 3). Other Pliocene generalist groups (beavers, gerbils and porcupines) appear to be scarce in Iberian faunas. More specialized groups (sciurids, glirids, cricetines and particularly murines) showed much lower number of species during the Pleistocene than in the Pliocene, although they did not show differences between their species BSI before and after the development of the first glaciations of the Pleistocene (Figure 3). These results corroborate the third prediction tested here.

## Discussion

The gradual decrease in the relative importance of specialist species during the Pliocene (increase in average BSI) is probably related to the progressive global cooling predominant during the Pliocene, which triggered modifications in the climate of southern Europe, from subtropical conditions with minor fluctuations of temperature to temperate conditions with noticeable annual thermal seasonality [49,50].

Our results are consistent with a scenario where species dwelling in more than one biome (generalists) were more able to tolerate this change due to their ability to find resources in different environments. After the development of the first modern glacial events at the Northern Hemisphere the specialization degree of the faunas progressively increased (the value of average BSI decreases). This trend reflects a gradual “recovery” of the rodent communities after the ecological disturbance caused by the onset of the first glaciations. Finally, during the Holocene the ecological specialization (average BSI) of the faunas reached similar values to the ones found during the early Pliocene.

**Figure 4:**
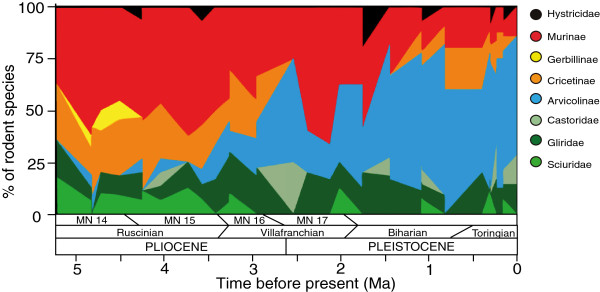
**Changes in the proportion in each rodent clade.** Variation of species during the Iberian Plio-Pleistocene.

There were profound changes in the faunal composition of the rodent assemblages (Figure 4) stemming from the development of the temperate climates of the Plio-Pleistocene [51]. During the Pliocene, Iberian rodent faunas were dominated by murines and cricetines, with a set of companion species within terrestrial squirrels and gerbils. In contrast, the dominant group during the Pleistocene was Arvicolinae (voles) [52]. Most of the correlations between the percentage of species in each family and the average BSI value of each fossil site before and after the modern glaciations are not significant (Table 3). Nevertheless, it is noteworthy that Arvicolinae shows a significant relationship with different sign in each period. Before 2.75 Ma, the moderate increase in the number of arvicoline species is associated with higher average BSI (more generalists). Yet, after the climatic crisis, the flowering of arvicoline faunas is associated to a significant decrease in average BSI of the rodent communities. This signifies that the increase in arvicoline diversity likely resulted from an adaptive radiation involving highly stenobiomic taxa. Furthermore, the radiation of voles had a preponderant role in the reorganization of the rodent faunas from the Iberian Plio-Pleistocene. During the Pliocene, this group is mainly represented by generalist species in *Promimomys*, *Dolomys* and *Mimomys*[34], and an important part of the survivors during the climatic “deterioration” of the middle-late Pliocene belong to the latter genus. During the Pleistocene the large radiation of arvicolines is dominated by the evolution of specialist species in many different genera, especially in *Microtus*[53,54]. Probably, this is related to the development of several new environments in the temperate latitudes [55], where resources were available for the exploitation by the generalist survivors of this family, which could start a process of progressive specialization along the Pleistocene. This is possibly one of the keys for the evolutionary success of this family (Figure 3), which now inhabits all the ecosystems of the Holarctic and is the dominant group of rodents in most of them.

**Table 3:** Correlation analyses between average BSI and the percentage of different rodent groups

	**Before 2.75 Ma**		**After 2.75 Ma**	
	**r**	p ^*^	**r**	p ^*^
**% Sciuridae**	−0.545	**0.036**	0.112	0.562
**% Gliridae**	0.346	0.206	0.052	0.790
**% Castoridae**	0.250	0.368	0.220	0.251
**% Arvicolinae**	0.711	**0.003**	−0.537	**0.003**
**% Cricetinae**	−0.054	0.849	0.095	0.625
**% Gerbillinae**	−0.628	**0.012**	(na) ^**†**^	(na) ^**†**^
**% Murinae**	−0.367	0.178	0.309	0.103
**% Hystricidae**	0.133	0.636	0.333	0.077

^*^ Bold, significant correlations.

^**†**^ na, not available (there are no species).

Coefficients and their significance for each group in the fossil sites before (N = 15) and after (N = 29) the onset of the Pleistocene glaciations at 2.75 Ma.

On the contrary, the other rodent groups of the Iberian Plio-Pleistocene were not able to take advantage of the new environments that were associated with the glacial-interglacial cyclicity of the Pleistocene climate. Particularly, in the case of Murinae there was a substantial decrease in the relative importance of this group in the rodent faunas from the Iberian Peninsula (Figure 4). From communities with representatives of eight genera during the Pliocene [56], the Pleistocene faunas only preserved species of *Apodemus*, *Castillomys*, *Micromys* and *Stephanomys*, the latter only surviving until 2.0 Ma [57,58]. Most murine species were adapted to forested and warm environments [34,42], which disappeared from Iberian latitudes with the onset of the modern glaciations. Today this family is predominantly distributed in tropical areas of the Old World, with only a few genera and species in temperate regions [59]. Murine demise in the Iberian Peninsula would be due not only to the decrease in temperature, but probably also to changes in precipitation amount and seasonality, associate to the development of Mediterranean climate [34]. Although such species loss was conspicuous in the Iberian Pleistocene, the specialization degree of this group did not show significant differences after 2.75 Ma (Figure 3).

Cricetinae was the other group showing a clear impoverishment over the analyzed interval. Whereas cricetid communities during the Pliocene held the genera *Apocricetus*, *Blancomys*, *Ruscinomys*, *Celadensia* or *Trilophomys*[60], Pleistocene cricetid faunas were only represented by the immigrant *Allocricetus bursae*. This taxon is closely related to the few genera that occupy today the arid environments present in the temperate regions of Eurasia since the onset of the glaciations. Apparently, the global cooling at the Plio-Pleistocene boundary imposed strong ecological limitations to the species adapted to arid and open environments, such as the Iberian cricetids [42,61]. Similarly, the terrestrial squirrels, characteristic of many Pliocene faunas from the Iberian Peninsula (*Atlantoxerus*), belong to Xerini, a group that today is restricted to the African savannas and semideserts, and disappeared completely from the Iberian record due to the cooling of the climate. They were replaced in the Iberian Pleistocene by Sciurini (*Sciurus*) and Marmotini (*Marmota*); these groups are predominant respectively in forested and open temperate environments. In the case of glirids, there were no large faunal changes before and after the beginning of the Pleistocene northern glaciations. They maintained the same genera, which are still living, and there are no significant differences in the BSI of their species over the period studied here (Figure 3).

Finally, three minor Pliocene generalist groups, gerbils, beavers and porcupines, were unable to proliferate in the Iberian Pleistocene. In the first case this is probably due to their specific specialization to arid climates mostly developed in other regions of Eurasia. The other two families show extremely low species number, which may have hampered their diversification.

It seems that the Pliocene witnessed a process of disassembly within the Iberian rodent communities. The impoverishment of the Iberian rodent communities throughout the Pliocene stemmed from a loss of diversity that affected most of the rodent families. The recovery of species diversity during the Pleistocene was linked to the radiation of stenobiomic arvicolines that resulted in the establishment of a new rodent fauna (Figure 4). This global radiation is evidenced within the Iberian fossil record by local speciation (e.g. development of the endemic lineage *Iberomys* within *Microtus*[62-64]) as well as by immigration of new species from other Eurasian regions, being both processes spurred by global environmental change. Overall, such ecological reorganization of assemblages appears to be triggered by global climatic changes and modulated by the differences in ecological specialization of the implied species, similarly to what was observed in earlier periods of faunal replacement in Spain [36,65].

## Conclusions

Our results offer support for some hypotheses included in Vrba’s habitat theory, which predicts a proportional decrease of specialist species associated with severe global climatic changes, and a later recovery of this kind of species associated to a complete faunal turnover. The pattern of faunal turnover is correlated with the development of the modern glaciations in the Northern Hemisphere around 2.75 Ma, which triggered a reorganization of the rodent communities as predicted by the turnover pulse hypothesis. In the same way our data support the resource-use hypothesis, which stresses the role of the degree of specialization in resources specifically related to particular biomes as a driver of differential speciation and extinction rates. In this way, ecological and evolutionary changes are intimately connected.

Finally, this work shows that the exceptional quality of the fossil record in the rodent assemblages from the Iberian Peninsula makes this group a good case for the study of the relevance of ecological characteristics of species in the development of macroevolutionary processes.

## Competing interests

The authors declare that they have no competing interests.

## Authors’ contributions

ARGC JLC and MHF conceived, designed and performed the data analyses, wrote the manuscript, co-edited all drafts and prepared the final version of the manuscript. AM and AMB co-edited early drafts. AM and MHF conducted the gathering of data and developed the idea for the manuscript. MHF conceived, designed and coordinated the study, initiated the project, facilitated the gathering of contributors, refined the intellectual content, coordinated the authorship survey and is the guarantor for the integrity of the article as a whole. All authors read and approved the final manuscript.
